# Book Reviews

**Published:** 1891-06

**Authors:** 


QUIZ COMPENDS, EQUINE ANAT. AND PHYS. By D. Ballon. Published by P. Blackiston, Son & Co.

This compilation is a condensation of Equine Anatomy and Physiology, is well illustrated. Every one who is interested in veterinary work would be pleased with this little book. It deals with an important subject, which is very much neglected.

KOCH'S REMEDY, IN RELATION TO THROAT CONSUMPTION. By Lennox Browne, London. Lea Brothers & Co., publishers, Philadelphia.

The treatment of tuberculosis in its various forms, by Koch's lymph, has been so thoroughly discussed and reported upon in the journals, that it would be unnecessary to enter into detail, suffice it to say that Mr. Browne has given in his characteristic way, his experience with the lymph, both in Germany and at home. The successful use of this remedy requires it to be in the hands of skillful diagnostitions, skilled in auscultation, opercussion and the use of the laryngoscope. It is claimed that the action of the remedy is dependant upon pathological cells and not upon the bacilli. Much judgment is required in selecting cases suitable, as success depends upon a limited area of tissue involved ; if this be extensive, the necrosis which follows, would overwhelm the system with poisonous ptomaines and dissolution would thus becourted. This book will be read with interest by all, especially those interested in throat troubles. P.

